# Valorization of Spent Coffee Grounds as a Natural Source of Bioactive Compounds for Several Industrial Applications—A Volatilomic Approach

**DOI:** 10.3390/foods11121731

**Published:** 2022-06-13

**Authors:** Carolina Andrade, Rosa Perestrelo, José S. Câmara

**Affiliations:** 1 CQM—Centro de Química da Madeira, Universidade da Madeira, Campus da Penteada, 9020-105 Funchal, Portugal; carolinafatimaandrade@hotmail.com (C.A.); rmp@staff.uma.pt (R.P.); 2 Departamento de Química, Faculdade de Ciências Exatas e Engenharia, Universidade da Madeira, Campus da Penteada, 9020-105 Funchal, Portugal

**Keywords:** spent coffee grounds, volatile fingerprint, circular economy, sustainability, industrial applications

## Abstract

Coffee is one of the most popular beverages worldwide, whose production and consumption result in large amounts of waste, namely spent coffee grounds, constituting an important source of compounds for several industrial applications. This work focused on the establishment of the volatile fingerprint of five spent coffee grounds from different geographical origins using headspace solid-phase microextraction coupled with gas chromatography-mass spectrometry (HS-SPME/GC-MS), as a strategy to identify volatile organic metabolites (VOMs) with potential application in the food industry as antioxidant, anti-inflammatory, and antiproliferative agents. One hundred eleven VOMs belonging to different chemical families were identified, of which 60 were found in all spent coffee grounds analyzed. Furanic compounds (34%), nitrogen compounds (30%), and esters (19%) contributed significant to the total volatile fingerprint. The data obtained suggest that spent coffee grounds have great potential to be used as raw material for different approaches in the food industry towards the development of new food ingredients or products for human consumption, in addition to pharmaceutical and cosmetic applications, namely as antioxidant (e.g., limonene, carvacrol), antimicrobial (e.g., pyrrole-2-carboxaldehyde, β-myrcene) and anti-inflammatory (e.g., furfural, 2-furanmethanol) agents, promoting their integral valorization within the circular bioeconomy concept.

## 1. Introduction

**Figure 1:**
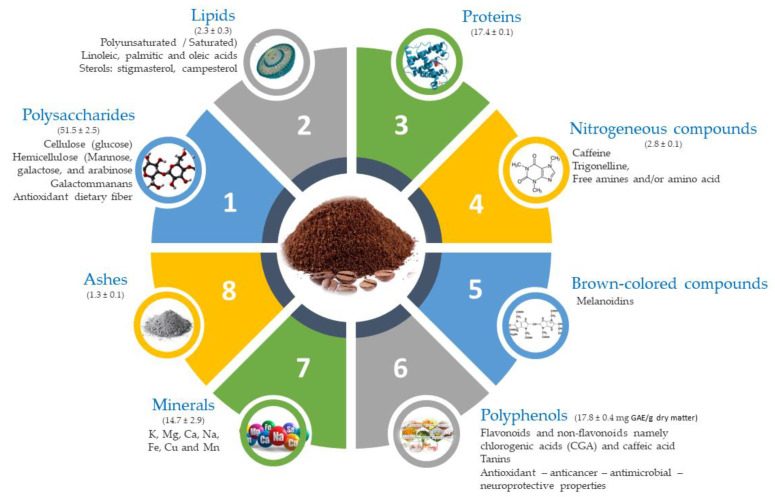
Main composition of spent coffee grounds (composition expressed in g/100 g of dry material).

Coffee (*Coffea* L.), Rubiaceae family, is one of the most popular beverages in the world with over 400 billion cups being consumed each year. A diversity of constituents including chlorogenic acids, pyrazines, furans, alkaloids, melanoidins (Maillard reaction product), among others, are responsible for their organoleptic characteristics. In addition, nutritional and health-promoting constituents including polyphenols (flavonoids, non-flavonoids), carotenoids, triterpenes, and high amounts of stimulant methylxanthine alkaloid, known as caffeine [1], and other molecules with functional properties, are part of coffee composition. As one of the most traded food products in the world after crude oil [2], coffee acts as a major source of income for many countries. In 2020, the International Coffee Organization estimated that coffee production surpassed 169 million 60 kg bags [3], with over 90% taking place in developing nations, namely in South America, while the consumption occurs primarily in industrialized economies. Despite the different flavors and varieties, there are only two types of coffee: (i) Coffea arabica var. Arabica, the most common and flavorful type, accounting for about 70% of world production; and (ii) *Coffea canefora* var. Robusta, is cheaper with a more aggressive flavor and contains higher amounts of antioxidants, caffeine, and soluble solids. Whereas green coffee is characterized by carbohydrates (60% dry weight), oligosaccharides, disaccharides (sucrose), and monosaccharides, the roasting leads to significant changes in the chemical composition due to the occurrence of the Maillard reaction, carbohydrate caramelization, and pyrolysis of organic compounds (Figure 1). In addition, a plethora of volatile organic metabolites (VOMs), important determinants of odor namely furanic compounds, volatile phenols, pyrazines, and aliphatic aldehydes, are produced and lack sustainable management.

Whilst coffee is an important economical asset, several millions of tons of waste are generated by the coffee chain, from the field to the cup. According to Brand et al. [4], only 6% by weight of the fresh coffee cherry harvested in the coffee field ends up in the cups. Hence, this leaves up to 94% as waste, which includes by-products from the process as well as water from the drying of the seeds [4], and spent coffee grounds, which comply to the largest contribution for coffee bio-waste, with approximately six million tons being produced yearly worldwide [5]. This type of waste is underutilized with large amounts ending in landfills which in turn can ultimately create serious environmental concerns [5], and constitutes a direct cost to producers, since it can lead to CO_2_ and other greenhouse gas emissions, and the release of chemicals such as caffeine, polyphenols, and tannins into the environment [2,6]. Additionally, similarly to other organic wastes, spent coffee grounds’ degradation involves great oxygen consumption due to its high carbon content [7], and there is an eminent risk of spontaneous combustion [5], heightening the need for its further valorization.

However, these natural low-cost by-products contain high amounts of bioactive components with high antiviral, antidiabetic, antioxidant, and neuroprotective potential [8,9,10]. This evidence, stimulate its exploitation as a renewable and sustainable bio-resource of added-value products with potential several applications, including in the food and pharmaceutical industry, regarding its valorization supported by the circular economy concept, as a powerful platform for shaping the future roadmap in the field of coffee chain sustainability. Moreover, these natural compounds are suitable candidates to replace the synthetic food additives associated with harmful effects on consumers including allergies, cardio- and cerebrovascular diseases and even cancer.

**Figure 2:**
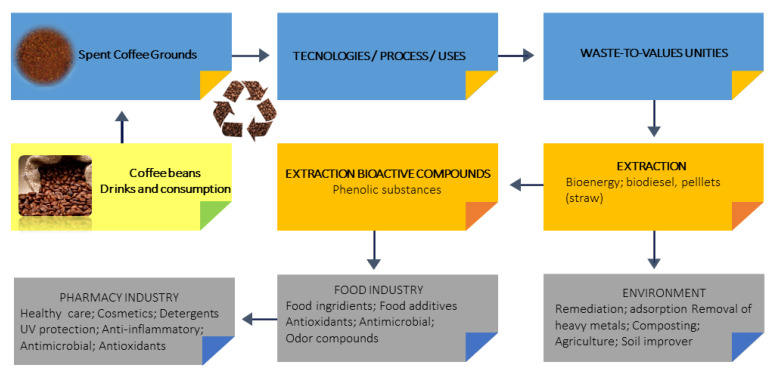
Overview of potential usages of spent coffee grounds.

Recent studies indicate that spent coffee grounds’ applications include its use to produce construction materials [8], as soil organic amendment, to improve soil fertility as it increases N, P, and K content [11,12], as a source of polysaccharides for the preparation of an adsorbent for the removal of uranium in water [13]. Spent coffee grounds could be also used as bug repellent, and for biodiesel and bioethanol production (Figure 2). However, scarce research has been done on the volatile fingerprint of spent coffee grounds. Therefore, there is an increasing interest in the characterization of specific volatile compounds in spent coffee grounds that might be used in the food industry as antioxidant, antimicrobial, and anti-inflammatory active agents for the formulation of novel functional foods with health benefits, meet the demand from consumers for healthy foods. In this context, this work focused on the establishment of the volatile fingerprint of different spent coffee grounds samples var. Arabica, using headspace solid-phase microextraction (HS-SPME) followed by gas chromatography-mass spectrometry (GC-MS), for the identification of valuable VOMs that could be used in the food industry for the development of new food ingredients or products for human consumption, pharmaceutical and cosmetic formulations, namely as antioxidant, antimicrobial and anti-inflammatory agents, promoting their integral valorization within the circular bioeconomy concept. Statistical tools were used to verify the differences between the volatile fingerprints of the spent coffee grounds.

## 2. Materials and Methods

### 2.1. Reagents and Standards

4-Methyl-2-pentanol (C_6_H_14_O, 99.0%) used as internal standard (IS) was acquired from Acros Organics (Geel, Belgium) and sodium chloride (NaCl, 99.5%) was supplied by Panreac (Barcelona, Spain). The SPME fiber holder for manual sampling together with 50/30 µm divinylbenzene/carboxen/polydimethylsiloxane (DVB/CAR/PDMS) fiber, with 1 cm length, was purchased from Supelco (Bellefonte, PA, USA). Fiber conditioning was performed daily according to the manufacturer’s recommendations in order to avoid carryover between sets of analyses. Helium (GC carrier gas) of purity 5.0 was supplied by Air Liquide (Lisbon, Portugal).

### 2.2. Sample Preparation

All samples were purchased from an Awaked company (Caldas da Rainha, Portugal). The roasted coffee samples were grounded until a thin powder was obtained. After that, 6 g of each sample were used to make 20 mL of expresso, in a coffee machine, and the spent coffee grounds were collected and stored in a flask for posterior analysis.

### 2.3. Headspace Solid-Phase Microextraction (HS-SPME) Procedure

The VOMs were extracted from the samples via HS-SPME. A total of 3 g of spent ground coffee sample, 1 g of NaCl, 3 mL of distilled water and 5 µL of 4-methyl-2-pentanol (IS), and a stirring bar were added to the extraction vial. The vial was then hermetically closed and submitted to the extraction temperature (50 °C) for 50 min, at a 450 rpm stirring rate, following the insertion of the DVB/CAR/PDMS fiber. All analysis was carried out in triplicate.

### 2.4. Gas Chromatography-Mass Spectrometry (GC-MS) Conditions

After the extraction was completed, the fiber holder was removed from the vial and inserted into the injector port of an Agilent Technologies 6890N Network gas chromatograph system (Palo Alto, California, USA), where the thermal desorption of the analytes was conducted at 250 °C during 6 min. The gas chromatographer was equipped with a 60 m × 0.25 mm I.D. × 0.25 µm film thickness HP-5 (SGE, Dortmund, Germany) fused silica capillary column and interfaced with an Agilent 5975 quadrupole inert mass selective detector. The oven temperature program was set as follows: initially set at 40 °C, increased until 220 °C at a rate of 2.5 °C/min, and maintained for 20 min at 220 °C. The total run time was 83 min. The column flow was constant at 1.0 mL/min using helium of purity 5.0. The injection port was operated in the splitless mode and held at 250 °C. For the 5975 MS system, the temperature of the quadrupole detector was 220 °C. Data acquisition was performed in the scan mode (30–300 *m*/*z*) with electron ionization at an energy of 70 eV. VOM identification was accomplished through manual interpretation, comparing the spectra with the data system library (NIST, 2005 software, Mass Spectral Search Program v.2.0d; NIST 2005, Washington, DC, USA) with a similarity threshold higher than 80%. The semi-quantification of VOMs was performed using 4-methyl-2-pentanol (IS) through the following equation: VOMs concentration = (VOM GC peak area/IS GC peak area) × IS concentration.

### 2.5. Statistical Analysis

Statistical analysis was carried out using MetaboAnalyst 5.0, which comprises the data pre-processing to eliminate VOMs with missing values (MV) and normalization (data transformation using data scaling by mean-center and cubic root). The normalized data was processed using the one-way ANOVA followed by Tukey’s test for post-hoc multiple comparisons of means and multivariate statistical analysis, including principal component analysis (PCA), an unsupervised method that was used to visualize group tendencies of the data set, using the information contained in the VOMs fingerprint as several variables to obtain insights into the separations among sample sets according to geographical origin, and partial least squares-discriminant analysis (PLS-DA), a supervised method performed using the relative peak area of the VOMs, that allows the investigation of the differences in the VOMs levels of spent coffee grounds and the identification of the VOMs that can be used to differentiate the spent coffee grounds samples. Finally, Pearson’s correlation was done to build the heat map of the spent coffee grounds using the VOMs identified with the purpose of recognizing clustering patterns.

## 3. Results

### 3.1. Volatile Fingerprint of Spent Coffee Grounds

The volatile fingerprint of the spent coffee grounds Arabica samples, from Guatemala, Colombia, Brazil, Timor, and Ethiopia, was determined using HS-SPME/GC-MS methodology, and the VOMs detected and identified are listed in Table 1, along with their relative concentration expressed in µg/L ± standard deviation for the different samples.

The GC-MS chromatograms of spent coffee grounds from different geographical origins were mapped in Figure S1 (Supplementary Materials).

A total of 111 VOMs was tentatively identified in the spent coffee grounds samples, 60 of which are common to all samples. These VOMs consisted of 29 nitrogen compounds, 20 carbonyl compounds, 16 furanic compounds, 11 terpenoids, 7 esters, 7 volatile phenols, 4 alcohols, 3 acids, 3 sulfur compounds, and 11 others.

**Figure 3:**
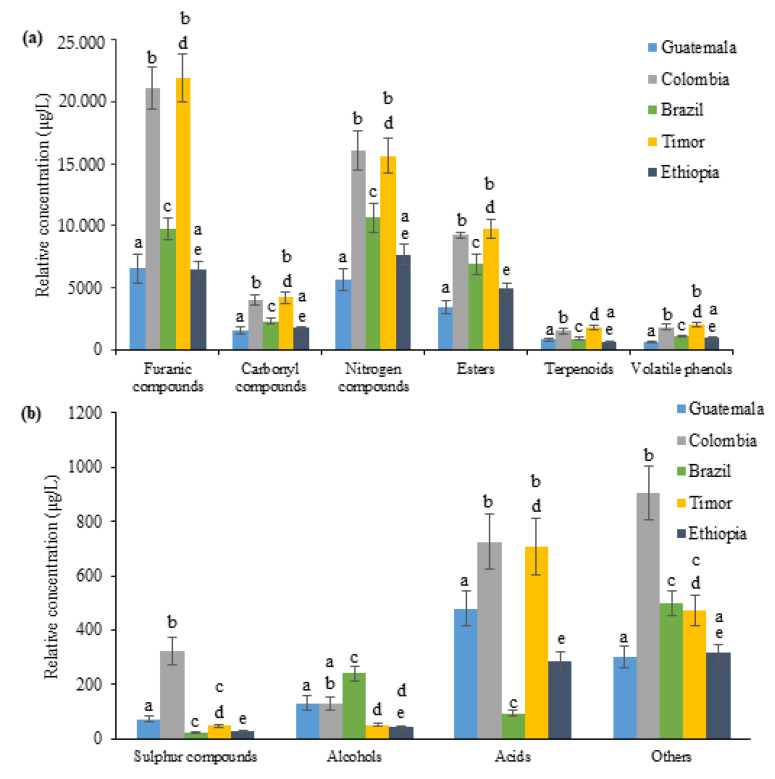
Relative concentration (µg/L) of the volatile organic metabolites identified grouped by major (**a**) and minor (**b**) chemical families for the spent coffee grounds. Different superscript letters in the same chemical family indicate significant differences (*p* < 0.05) among spent coffee grounds from different geographical origins.

In semi-quantitative terms, furanic compounds (13,150 ± 903.0 μg/L for total relative concentration of volatile fingerprint), nitrogen compounds (11,125 ± 733.3 μg/L), esters (6862 ± 426.5 μg/L), carbonyl compounds (2772 ± 122.3 μg/L), volatile phenols (1310 ± 85.6 μg/L) and terpenoids (1122 ± 89.6 μg/L) were the chemical families with the highest contribution to the volatile fingerprint of spent coffee grounds. The contribution of the remaining chemical families to the total volatile fingerprint was lower than 2%. Figure 3 shows the distribution of the VOMs, according to chemical family. Significant statistical differences were observed (Figure 3) in terms of relative concentration among chemical families identified in spent coffee grounds from different coffee investigated samples.

Furanic and nitrogen compounds found in coffee are mainly products of the Maillard reaction as well as thermal and Strecker degradations of their precursors (carbohydrates and proteins) [14], which occur during the roasting of the coffee beans. Furan compounds have been shown to have potential as alternative commodity chemicals to fossil-fuel-based platform chemicals, through the oxidation, dehydration, and hydrogenation process of functionalities attached to their furan ring [15], producing a variety of value-added chemicals [16]. Spent coffee grounds from Timor seem to be the richest in furanic compounds (21,953 ± 1373 μg/L), followed by spent coffee grounds produced in Colombia (21,091 ± 1298.9 μg/L), Brazil (9722 ± 840.5 μg/L), Guatemala (6531 ± 919.9 μg/L) and Ethiopia (6453 ± 517.1 μg/L).

Furfural (2359 ± 224.9 μg/L of the total relative concentration of volatile fingerprint), 5-methylfurfural (4391 ± 415.6 μg/L) and 2-furanmethanol (3339 ± 311.3 μg/L) were the most abundant furanic compounds identified in all spent coffee grounds. Furfural can be transformed into useful fuels and chemicals used in oil refining, plastics production, and the pharmaceutical and agrochemical industries [17]. This furanic compound was identified in all the spent coffee ground samples analyzed in high relative concentrations but was more abundant in spent coffee grounds from Colombia and Timor, Table 1. On the other hand, benzofuran was only detected in spent coffee grounds from Brazil, whereas vinyl furan was not detected only in spent coffee grounds from Guatemala.

Nitrogen compounds represent the second most abundant chemical group identified in the analyzed samples, of which the majority consisted of pyrazines and pyrroles. Pyrazine and its derivatives have various applications as ingredients in pesticides, insecticides, dyes, pharmaceutical products, corrosion inhibitors, organic photovoltaics, and organic light emitting diodes [18]. Moreover, alkylated pyrazines are used as flavoring agents in the food industry due to their strong aromatic properties, while methoxylated pyrazines in the perfume industry are used to improve the odor of cosmetics and toiletries [19]. On the other hand, pyrrole and its derivatives have been proved to play a significant role in material science, as a component of optoelectronic equipment, and in pharmaceutical chemistry, since several pyrrole-based drugs have been discovered [20]. The nitrogen compounds’ contribution to the total volatile fingerprint is similar between the spent coffee grounds from Ethiopia and Brazil, 33.27 and 32.72%, respectively. Nevertheless, this chemical family has a less contribution to the total volatile fingerprint in spent coffee grounds from Colombia (28.81%), Guatemala (28.74%) and Timor (27.58%). 2-Ethyl-6-methylpyrazine (941.2 ± 103.4 μg/L of the total relative concentration of volatile fingerprint), 3-ethyl-2,5-dimethylpyrazine (960.4 ± 83.11 μg/L) and 1-(2-furanylmethyl)pyrrole (1150 ± 90.24 μg/L) were the most abundant nitrogen compounds in spent coffee grounds independently of geographical origin.

Esters, synthesized through the oxidation of fatty acids, contribute to the fruity and floral flavor of spent coffee grounds, and depending on their specific properties have a wide range of applications in the cosmetic (e.g., emollients in creams, surfactants in shampoos, antioxidants in antiaging creams, fragrances in perfumes) (Khan and Rathod, 2015) and food (e.g., flavor and aroma of diversity of food-related products) industry [21]. Spent coffee grounds from Timor seem to be the richest in esters (9749 ± 599.0 μg/L), followed by spent coffee grounds produced in Colombia (9237 ± 195.5 μg/L), Brazil (6915 ± 652.3 μg/L), Guatemala (3426 ± 436.0 μg/L) and Ethiopia (4985 ± 249.8 μg/L). 2-Furfuryl acetate (5973 ± 456.6 μg/L of the total relative concentration of volatile fingerprint) was the predominant esters identified in all spent coffee grounds independently of geographical origin.

Carbonyl compounds (aldehydes and ketones) were a similar contribution to the total volatile fingerprint among the spent coffee grounds from Guatemala (7.97%), Ethiopia (7.64%), Timor (7.43%), Colombia (7.18%) and Brazil (7.10%). 1-Methylpyrrole-2-carboxaldehyde (605.4 ± 66.74 μg/L of the total relative concentration of volatile fingerprint) and 1-(acetyloxy)-2-propanone (529.7 ± 42.76 μg/L) were the most abundant carbonyl compounds identified in spent coffee grounds analyzed. 2-Methylpropanal and 3-hexanone were not detected in spent coffee grounds from Guatemala, 2-methylcyclopent-2-en-1-one and 2,3,4-trimethylcyclopent-2-en-1-one in spent coffee grounds from Ethiopia, and 3-ethyl-2-hydrodoxy-2-cyclopenten-1-one in spent coffee grounds from Brazil. Terpenoids are compounds produced by plants (phytochemicals) and are one of the largest classes of natural compounds, owing to their chemical diversity. Therefore, they can have a wide range of industrial applications, such as flavors, fragrances, high-grade lubricants, biofuels, agricultural chemicals, and medicines [22]. The terpenoids contribution to the total volatile fingerprint is similar among the spent coffee grounds from Brazil (2.83%), Colombia (2.70%) and Ethiopia (2.46%), being highest in spent coffee grounds from Guatemala (4.11%). Carvacrol (653.6 ± 83.32 μg/L of the total relative concentration of volatile fingerprint), limonene (153.8 ± 15.45 μg/L) and 2-menthene (125.0 ± 12.56 μg/L) were the most abundant terpenoids in spent coffee grounds independently of geographical origin. Carvacrol has been progressively employed in the food and pharmaceutical industries due to its strong antibacterial and anti-inflammatory properties [23]. Nevertheless, carvacrol is volatile, easily oxidized, and difficult to dissolve in water, and for this reason, several studies have been developed to improve its stability through encapsulation methods [16,23]. Limonene was used as a green and non-toxic solvent alternative to produce polystyrene fiber matrix by electrospinning [16] and applied in the agri-food industry as an antimicrobial, herbicidal, and antioxidant agent [24].

### 3.2. Potential Applications of Spent Coffee Grounds Volatile Compounds

In nature, VOMs, perform numerous functions for the communication between insects and/or plants for (insect) mating or even, because of their pleasant smell or taste, as fragrances and flavors. In fruits and vegetables [25], these secondary metabolites are generated from fatty acids or amino acid precursors during ripening, whiles in other foods several pathways can explain its formation such as the levurian metabolism in fermented beverages, the oxidation/reduction processes during storage/aging, the Maillard and Strecker reactions which occurs as a result of some thermal procedures to which some foods are subjected, the coffee roasting and wine heating (*estufagem* in Madeira wine), for instance, with the formation of compounds that impact the aroma of different products such as furan, sulfur, and nitrogen-containing compounds [26].

There is increasing demand for natural compounds which can act as food preservatives to inhibit the growth of microorganisms (Table 2), especially pathogenic microorganisms, and to control the natural spoilage process. In this context, the use of waste from the agri-food industry constitutes an important natural, cheap, and renewable source of compounds with a potential preservative effect, among which, in addition to phytochemicals, a wide variety of VOMs can be highlighted [27].

Some of the VOMs identified in the spent coffee grounds analyzed have been pointed out for presenting biological activity, including antidiabetic, anti-inflammatory, antimicrobial, antioxidant, antiproliferative, and antitumor activities, potentiating their applications in the food industry as preservatives, additives, flavoring agents, among others (Table 2) [25,27,28,29,30,31,32,33,34,35]. A semi-quantitative analysis on volatile profile of spent coffee ground was performed by Page et al. [36], The most abundant chemical classes identified in the evaluated samples were furan compounds, pyrrols, phenolics, and carboxylic acids. Terpenes, alcohols, ketones, aldehydes, thiophenes, tiazoles, and benzoxazoles were also identified in minor quantities. In addition, Zambonin et al. [37] also identified pyrazines, furans, pyrrols, and phenolic compounds as the most abundant classes of compounds when studying coffee brews using HS-SPME/GC–MS.

Ten of these VOMs were indicated to have antimicrobial activity which were furfural [33], 5-methylfurfural [35], 2-furanmethanol [28], pyrrole-2-carboxaldehyde [30], hexanal, β-myrcene, limonene, guaiacol [25], carvacrol [34], and menthene [29]; eight compounds with antioxidant activity, including furfural, 2-furanmethanol [27], β-myrcene, limonene, guaiacol [25], carvacrol [34], menthene [29], and dimethyl disulfide [31]; five compounds with anti-inflammatory activity including furfural, 2-furanmethanol [27], limonene [25], carvacrol [34], and menthene [29]; three compounds with antitumor activity 5-methylfurfural [35], limonene [25], and carvacrol [34]; three compounds that present cytotoxic activity, including β-myrcene, limonene [25], and carvacrol [34]; and one compound, limonene [25], with antidiabetic activity. Additionally, three of the identified VOMs were used as flavoring agents, including 5-methylfurfural [35], 2-ethyl-6-methylpyrazine, and 3-ethyl-2,5-dimethylpyrazine [32].

These VOMs properties enhance its potential use in different areas including the food, pharmaceutical, and cosmetics industry, valuing a natural, renewable, and low-cost raw material, from a circular economy perspective.

### 3.3. Statistical Analysis

**Figure 4:**
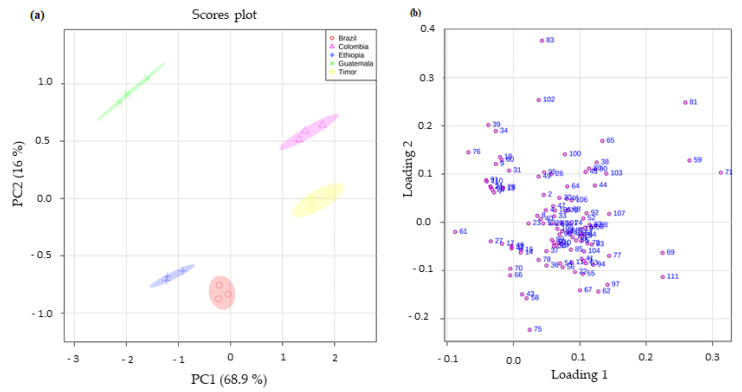
PCA of the volatile fingerprint of spent coffee grounds from different geographical origin. (**a**) PC1 × PC2 score scatter plot and (**b**) loading weight plot (attribution of the peak number is shown in Table 1).

The one-way ANOVA with post-hoc Tukey test (*p* < 0.05) performed showed that, the *p* values obtained proved that the identified 111 VOMs presented statistically significant differences for the analyzed spent coffee grounds from different geographical origins. Statistical analysis was applied to the spent coffee grounds through the principal component analysis (PCA) multivariate pattern recognition procedure, to determine the differences in the volatile fingerprint. Figure 4a,b present the PCA score plot and loading plot from the spent coffee grounds from different geographical origins, respectively. All spent coffee grounds were adequately separated, underlining that the total 111 VOMs identified in the five different samples allowed their discrimination.

**Figure 5:**
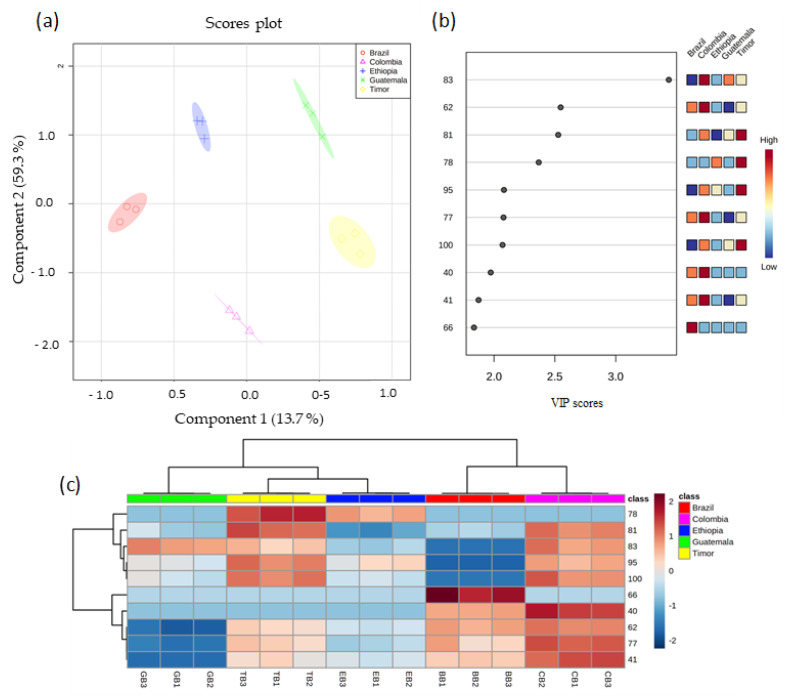
PLS-DA of the volatile fingerprint of spent coffee grounds from different geographical origins (**a**) score scatter plot, and (**b**) VIP scores and (**c**) hierarchical cluster analysis (HCA). (Attribution of the peak number is shown in Table 1).

The variances of PC1 and PC2 were 68.9 and 16%, respectively, representing 84.9% of the total variability of data. Colombia spent coffee grounds, projected in PC1 and PC2 positive, was characterized by 2-furanmethanol (81), 2-acetylfuran (65), and furfural (59), whereas the spent coffee grounds from Ethiopia and Brazil projected in PC1 and PC2 negative by trimethyloxazole (27), methyl-2-butanoate (17), 1-octanol (70), 2-methylbenzofuran (75), 2-methyl-5-propylpyrazine (58), and 2-heptanol (43). Guatemala spent coffee grounds, projected in PC1 negative and PC2 positive, was characterized by caryophyllene (76), o-cymene (39), and γ-terpinene (34), while Timor spent coffee grounds placed in PC1 positive and PC2 negative by 2-furfuryl acetate (69), carvacrol (111), and 5-methylfurfural (71). Moreover, partial least square-discriminant analysis (PLS-DA) was applied (Figure 5) and the 10 most significant VOMs (VIP score > 1.5) that allowed the discrimination of the spent coffee ground samples by geographical origin were 3-methylbutanoic acid (83), 3,5-diethyl-2-methylpyrazine (62), 2-furanmethanol (81), 2-acetylpyridine (78), 3-methyl-2-butenoic acid (95), 2,2’-methylenebisfuran (77), 3-ethyl-2-hydroxy-2-cyclopenten-1-one (100), 2,7-dimethyloxepine (40), 3-methylanisole (41), and benzofuran (66) (Figure 4b). 

A random permutation test with 1000 permutations was performed with PLS-DA model (Figure 5) to evaluate the robustness of the model.

The heatmap created using Pearson’s correlation for the VOMs with VIP scores > 1.5 is shown in Figure 5c. The heatmap shows that most of these VOMs presented higher chromatographic area in Colombia spent coffee grounds, except for 2-acetylpyridine (78) and benzofuran (66), that presented higher values in Timor and Brazil spent coffee grounds, respectively. In addition, as seen in Figure 5c, the volatile fingerprint of spent coffee grounds showed remarkable differences among the geographical regions. This variance could be an outcome of climate conditions factors (e.g., mean annual temperature), altitude and geographic location (e.g., longitude, latitude), which may result in an inhibition of the activity of certain odor-related enzymes [38,39].

**Figure 6:**
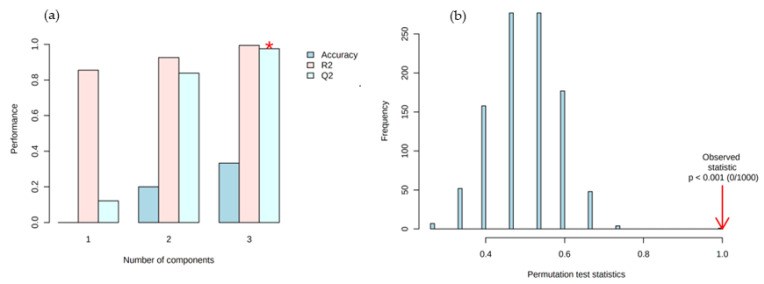
(**a**) 10-fold cross-validation performance and (**b**) model validation by permutation test based on 1000 permutations of VOCs obtained by GC-MS of spent coffee grounds samples (* means best Q2 value).

In Figure 6a goodness of fit of 0.9944 (R2 = 99.44%) and a predicted ability of 0.9813 (Q2 = 98.13%) were obtained with three significant components, based on crossing-validation. The model is not overfitted, as can be proved by the difference between R2 and Q2, which was lower than 0.3 (R2 − Q2 = 0.01). This indicates that it presented an acceptable predictive ability for the discrimination of the spent coffee ground from different geographical origin.

## 4. Conclusions

The volatile fingerprint of spent coffee grounds from different lots of coffee was established using HS-SPME/GC-MS to explore their potential to be used for industrial applications, to valorize this major coffee by-product. A total of 111 VOMs belonging to different chemical families was identified in the spent coffee grounds from different geographic origins, 60 of which were common to all samples. Furanic compounds (accounts for 34% of the total volatile fingerprint), nitrogen compounds (30%), and esters (19%), were the chemical families with the highest contribution to the volatile fingerprint of spent coffee grounds, followed by carbonyl compounds (7%), volatile phenols (4%), and terpenoids (3%). The contribution of the remaining chemical families (alcohols, sulphur compounds, acids) for the total volatile fingerprint was lower than 2%. In addition, remarkable differences in terms of the qualitative and semi-quantitative profiles were observed among the spent coffee grounds analyzed suggesting that geographical origin and climatic conditions have a substantial impact on the volatile fingerprint. A clear discrimination of spent coffee grounds from different geographical origin was observed, giving three furanic compounds, one carbonyl compound, two nitrogen compounds, and two acids as putative geographical markers.

In sum, the most abundant chemical families (furanic compounds, nitrogen compounds, esters, terpenoids) identified in the analyzed spent coffee grounds showed great potential to be used as raw material for applications in food (as an ingredient and/or value-added components) and/or in non-food products (e.g., pharmaceuticals, cosmetics, food packing), supporting its valorization based on circular economy concept closing the loop of coffee value chain, toward the valorization of coffee by-product. The outcome of this research work represents a noteworthy contribution to circular economy, as it confirms spent coffee grounds can be used for a diversity of industrial applications and have high availability throughout the year, in addition to its low price. The evaluation of the bioactive potential of spent coffee grounds is being evaluated in terms of antioxidant, antiproliferative and antihypertensive is being carried out in addition to the identification of the polyphenols responsible for the related activity. The isolation of the bioactive compounds followed by their nanoencapsulation will be a strategy for its application in pharmaceutical formulations. In addition, future works to produce natural flavoring products for deployment in the food and beverage industry, are under consideration.

## Figures and Tables

**Table 1:** Relative concentration (µg/L) of volatile organic compounds in the spent coffee grounds from different geographical origins using HS-SPME/GC–MS.

Peak n°	RT (min)	Chemical Families	Relative Concentration (µg/L) ± Standard Deviation				
			Guatemala	Colombia	Brazil	Timor	Ethiopia
*Furanic compounds*							
3	10.69	2-Methylfuran	32.35 ± 2.807	165.8 ± 18.27	157.6 ± 3.476	279.3 ± 35.10	81.75 ± 11.61
8	13.679	2,5-Dimethylfuran	32.45 ± 3.151	67.83 ± 10.29	45.82 ± 3.605	59.84 ± 7.554	30.22 ± 2.647
11	19.48	Vinylfuran	-	68.22 ± 10.09	62.12 ± 3.754	80.80 ± 14.44	25.15 ± 0.591
29	27.45	2-(2-Propenyl)furan	57.81 ± 9.025	174.4 ± 12.13	109.3 ± 1.837	128.0 ± 24.93	78.47 ± 14.94
31	28.67	2-Penthylfuran	153.7 ± 27.17	123.1 ± 6.383	81.27 ± 3.392	72.71 ± 11.59	50.92 ± 6.991
33	29.18	2-(Methoxymethyl)furan	40.75 ± 4.517	130.8 ± 4.067	64.74 ± 10.38	114.1 ± 11.85	55.75 ± 0.328
59	42.77	Furfural	1052 ± 185.5	3846 ± 303.1	1537 ± 195.4	4317 ± 344.7	1037 ± 95.63
65	45.12	2-Acetylfuran	611.6 ± 80.38	1319 ± 79.80	517.9 ± 38.71	1262 ± 100.6	360.5 ± 42.45
66	45.31	Benzofuran	-	-	118.5 ± 19.35	-	-
71	48.89	5-Methylfurfural	1876 ± 365.1	7150 ± 635.8	3071 ± 98.47	7625 ± 788.2	2235 ± 191.8
73	49.98	2,2’-Bifuran	68.59 ± 6.937	406.7 ± 38.41	233.6 ± 78.03	321.8 ± 6.142	167.6 ± 26.19
75	50.26	2-Methylbenzofuran	-	-	139.0 ± 19.08	101.6 ± 6.604	61.61 ± 6.493
77	50.47	2,2’-Methylenebisfuran	205.6 ± 15.90	1205 ± 40.91	777.4 ± 149.7	747.6 ± 56.68	442.8 ± 38.63
81	52.74	2-Furanmethanol	2131 ± 408.7	5270 ± 382.1	2096 ± 118.6	5916 ± 464.1	1280 ± 183.2
84	53.98	2-Furfuryl-5-methylfuran	153.0 ± 29.48	688.7 ± 60.06	380.1 ± 65.47	447.8 ± 23.57	294.8 ± 17.45
104	68.38	Difurfuryl ether	116.2 ± 13.61	471.7 ± 63.78	330.5 ± 50.35	479.4 ± 49.76	251.1 ± 32.65
*Carbonyl compounds*							
1	9.21	2-Methylpropanal	-	39.23 ± 5.756	20.99 ± 2.912	42.71 ± 3.692	15.65 ± 2.433
2	9.27	Acetone	36.77 ± 6.790	47.40 ± 3.951	25.49 ± 3.514	88.42 ± 12.57	18.87 ± 1.544
4	11.65	2-Butanone	18.21 ± 1.393	48.25 ± 6.338	23.08 ± 2.668	53.50 ± 4.322	18.04 ± 1.360
5	12.12	2-Methylbutanal	37.91 ± 6.963	185.7 ± 29.20	98.24 ± 8.734	280.1 ± 25.59	101.5 ± 11.28
6	12.27	3-Methylbutanal	19.09 ± 3.443	101.6 ± 13.12	48.51 ± 3.580	128.3 ± 0.579	47.23 ± 2.425
10	18.33	3-Hexanone	-	50.66 ± 7.312	26.30 ± 2.368	32.27 ± 0.898	16.48 ± 1.638
13	20.00	Hexanal	161.6 ± 21.45	76.59 ± 10.58	123.2 ± 4.822	84.80 ± 16.42	35.43 ± 1.386
15	20.85	2-Methyl-2-butenal	-	34.98 ± 2.586	-	49.81 ± 7.886	20.91 ± 2.539
49	37.46	2-Methylcyclopent-2-en-1-one	30.82 ± 4.513	24.43 ± 2.215	15.80 ± 1.861	51.17 ± 4.746	-
57	42.30	1-(Acetyloxy)-2-propanone	262.7 ± 50.82	772.4 ± 44.97	485.1 ± 14.64	742.4 ± 66.74	386.0 ± 36.64
64	44.76	2,3,4-Trimethylcyclopent-2-ene-1-one	47.01 ± 4.102	97.00 ± 14.88	69.10 ± 7.897	133.5 ± 23.56	-
68	46.10	1-(Acetyloxy)-2-butanone	69.79 ± 8.213	192.8 ± 11.79	98.62 ± 7.483	239.5 ± 38.96	78.67 ± 5.236
80	51.66	1-Methylpyrrole-2-carboxaldehyde	409.1 ± 60.99	1009 ± 96.13	408.2 ± 44.55	863.8 ± 125.8	337.4 ± 6.290
85	54.50	1-(5-Methyl-2-furanyl)-1-propanone	51.11 ± 8.913	193.3 ± 19.20	161.9 ± 15.62	245.7 ± 18.78	104.3 ± 3.237
88	55.44	3-Thiophenecarboxaldehyde	81.95 ± 6.049	194.1 ± 13.32	128.1 ± 4.330	230.0 ± 12.46	110.1 ± 3.956
89	55.77	4-(5-Methyl-2-furanyl)-2-butanone	55.04 ± 7.713	150.9 ± 9.911	117.7 ± 14.19	168.3 ± 13.30	99.93 ± 1.155
96	60.67	3,4-dimethylpyrrole-2-carboxaldehyde	79.86 ± 15.15	238.2 ± 23.10	163.8 ± 24.02	229.2 ± 28.86	133.4 ± 4.687
100	64.50	3-Ethyl-2-hydroxy-2-cyclopenten-1-one	48.21 ± 10.05	140.3 ± 27.11	-	142.8 ± 11.36	45.20 ± 3.894
101	65.20	4-(2-Furanyl)-3-buten-2-one	75.43 ± 12.29	222.4 ± 5.494	154.6 ± 18.53	197.4 ± 30.26	89.65 ± 16.57
109	73.79	1-Methylpyrrole-2-carboxaldehyde	73.36 ± 11.76	186.6 ± 24.95	137.8 ± 22.56	207.6 ± 25.33	98.89 ± 7.737
*Nitrogen compounds*							
19	23.48	1-Methylpyrrole	-	56.41 ± 9.041	43.53 ± 1.601	113.8 ± 17.90	-
25	25.89	Pyridine	231.5 ± 16.62	465.3 ± 17.32	549.1 ± 52.99	672.2 ± 104.2	180.8 ± 31.07
30	27.77	Pyrazine	19.67 ± 1.841	47.68 ± 5.511	35.46 ± 4.561	67.02 ± 8.104	-
38	30.99	Methylpyrazine	594.3 ± 66.29	1206 ± 62.03	621.6 ± 80.05	1252 ± 100.3	375.4 ± 51.81
44	34.31	2,5-Dimethylpyrazine	4870 ± 74.89	1228 ± 89.84	631.7 ± 77.06	1075 ± 106.1	446.4 ± 58.79
45	34.68	2,6-Dimethylpyrazine	534.2 ± 75.54	1157 ± 81.97	577.6 ± 86.41	989.7 ± 94.39	412.0 ± 58.61
46	35.09	Ethylpyrazine	324.31 ± 26.88	623.1 ± 82.54	405.2 ± 66.44	610.5 ± 55.32	279.3 ± 31.50
47	35.80	2,3-Dimethylpyrazine	100.6 ± 16.08	193.4 ± 6.569	135.2 ± 20.53	201.6 ± 12.12	76.31 ± 11.12
48	37.33	1-Pentylpyrrole	-	-	12.25 ± 0.496	-	-
50	38.03	2-Ethyl-6-methylpyrazine,	495.6 ± 84.41	1396 ± 104.9	860.8 ± 130.4	1216 ± 113.5	737.2 ± 83.66
51	38.42	2-Ethyl-5-methylpyrazine	381.5 ± 70.70	1056 ± 127.9	695.6 ± 68.88	906.9 ± 108.8	590.8 ± 53.03
52	39.13	Trimethylpyrazine	390.7 ± 54.93	1029 ± 123.9	644.4 ± 72.31	897.8 ± 95.26	497.1 ± 36.64
53	40.03	2-(n-Propyl)pyrazine	44.63 ± 7.261	80.94 ± 3.038	71.45 ± 10.83	105.7 ± 12.70	44.33 ± 3.517
54	40.84	2,6-Diethylpyrazine	91.39 ± 13.22	1329 ± 27.96	274.7 ± 25.70	280.7 ± 28.03	233.4 ± 21.70
55	41.37	3-Ethyl-2,5-dimethylpyrazine	413.5 ± 62.74	1329 ± 150.5	1086 ± 55.40	1079 ± 80.68	894.1 ± 66.24
56	41.96	2,3-Dimethylpirazine	-	88.19 ± 7.728	77.43 ± 2.614	90.05 ± 15.41	53.59 ± 3.506
58	42.56	2-Methyl-5-propylpyrazine	-	-	54.63 ± 0.121	86.75 ± 9.378	67.38 ± 4.048
61	44.02	2,3-Diethyl-5-methylpyrazine	51.43 ± 6.440	-	-	-	112.7 ± 9.891
62	44.09	3,5-Diethyl-2-methylpyrazine	77.78 ± 13.28	277.9 ± 35.88	571.2 ± 35.19	423.6 ± 31.38	290.9 ± 12.74
63	44.49	2-Ethenyl-6-methylpyrazine	-	156.5 ± 18.49	107.1 ± 5.82	170.3 ± 32.77	66.40 ± 1.476
67	45.82	2-Methoxy-3-(2-methylpropyl)pyrazine	-	-	258.5 ± 30.03	528.2 ± 48.95	-
74	50.07	Isopropenylpyrazine	40.46 ± 8.075	186.7 ± 20.50	79.01 ± 12.64	178.6 ± 20.04	83.51 ± 11.45
78	50.56	2-Acetylpyridine	-	-	-	134.0 ± 19.05	69.45 ± 8.223
82	53.22	2-Acetyl-1-methylpyrrole	253.6 ± 48.89	847.5 ± 99.88	534.5 ± 44.33	683.6 ± 21.41	337.4 ± 33.81
87	55.22	2-Acetyl-3-methylpyrazine	63.38 ± 8.495	237.2 ± 35.77	136.8 ± 9.104	252.8 ± 24.67	114.8 ± 9.090
90	56.25	3-Methylpyrrole	53.57 ± 7.528	204.2 ± 22.15	87.33 ± 12.65	213.9 ± 26.46	-
97	61.57	1-(2-Furanylmethyl)pyrrole	403.7 ± 77.24	1615 ± 89.00	1291 ± 108.4	1462 ± 92.84	977.4 ± 83.77
103	67.99	2-Acetylpyrrole	263.2 ± 52.03	803.2 ± 156.7	291.3 ± 36.40	837.9 ± 73.19	234.3 ± 37.07
107	70.52	Pyrrole-2-carboxaldehyde	304.7 ± 55.57	1081 ± 188.2	502.1 ± 76.74	1102 ± 44.02	483.3 ± 75.45
*Esters*							
17	21.41	Methyl-2-butanoate	-	-	-	-	15.76 ± 1.038
32	28.77	Undecyl benzoate	-	-	13.51 ± 1.205	-	-
36	30.00	3,3-Dimethylallyl acetate	-	37.87 ± 2.202	46.03 ± 9.062	47.30 ± 1.309	32.02 ± 0.934
69	46.38	2-Furfuryl acetate	3090 ± 497.2	7930 ± 144.8	6097 ± 737.2	8499 ± 633.6	4250 ± 270.1
72	49.57	2-Furfuryl propanoate	205.4 ± 29.76	638.7. ± 22.90	438.7 ± 33.69	504.0 ± 55.12	328.2 ± 39.06
86	54.54	Furfuryl 3-methylbutanoate	89.78 ± 15.27	379.5 ± 46.98	198.8 ± 39.16	354.2 ± 23.96	224.8 ± 6.479
93	59.60	Methyl-2-hydroxybenzoate	40.45 ± 6.367	251.1 ± 38.54	121.0 ± 17.56	344.2 ± 40.12	133.9 ± 8.486
*Terpenoids*							
18	22.06	β-Terpinene	28.99 ± 1.445	17.29 ± 2.617	-	-	-
21	23.74	3-Carene	23.20 ± 3.721	-	-	-	-
23	24.43	β-Myrcene	26.69 ± 3.975	58.45 ± 9.665	37.32 ± 2.044	39.95 ± 5.829	38.64 ± 1.532
24	25.19	α-Terpinene	26.13 ± 3.925	-	-	-	-
28	26.80	Limonene	194.1 ± 25.74	200.0 ± 16.20	103.3 ± 9.771	106.9 ± 16.01	164.8 ± 9.548
34	29.65	γ-Terpinene	87.64 ± 11.33	54.32 ± 6.037	-	-	-
35	29.83	β-cis-Ocimene	-	68.74 ± 6.549	34.20 ± 1.713	34.79 ± 5.172	30.30 ± 3.454
39	31.30	Cymene	107.0 ± 17.59	51.05 ± 7.566	-	-	-
76	50.35	Caryophyllene	172.7 ± 33.19	-	-	-	-
92	59.16	2-Menthene	40.14 ± 6.087	199.9 ± 21.73	78.83 ± 11.04	244.1 ± 15.15	62.24 ± 8.815
111	77.92	Carvacrol	96.52 ± 14.20	855.3 ± 124.3	653.5 ± 126.8	1393 ± 118.5	270.3 ± 32.90
*Volatile phenols*							
22	24.05	3-Methylphenol	-	156.2 ± 17.66	126.1 ± 10.80	113.0 ± 1.997	66.73 ± 9.176
98	63.00	Guaiacol	263.7 ± 41.87	852.5 ± 118.3	457.4 ± 26.62	903.4 ± 83.59	441.3 ± 36.06
99	63.93	*o*-Cresol	85.86 ± 15.10	222.8 ± 36.49	178.5 ± 9.106	231.5 ± 19.46	151.9 ± 17.41
105	69.00	*p*-Cresol	63.46 ± 5.820	141.5 ± 3.574	82.15 ± 3.970	167.8 ± 12.53	63.80 ± 9.344
106	69.26	Phenol	119.9 ± 19.63	287.0 ± 28.80	142.9 ± 8.188	345.9 ± 32.88	117.3 ± 20.18
108	72.78	*m*-Cresol	61.88 ± 5.393	164.9 ± 27.65	131.1 ± 20.10	281.9 ± 39.97	84.74 ± 12.16
110	76.46	*m*-tert-Butylphenol	43.97 ± 0.994	-	-	-	-
*Sulphur compounds*							
12	19.65	Dimethyldisulfide	-	33.51 ± 5.493	-	47.00 ± 6.383	15.04 ± 0.262
14	20.67	2-Methylthiophene	-	21.62 ± 0.893	21.65 ± 1.543	-	13.39 ± 0.668
102	66.71	2-Thiophenemethanol	72.11 ± 10.73	266.0 ± 46.03	-	-	-
*Alcohols*							
42	33.30	3-Methylbenzyl alcohol	-	-	15.21 ± 1.480	-	-
43	33.44	2-Heptanol	-	-	49.17 ± 8.216	48.71 ± 5.321	40.74 ± 4.082
60	43.31	2-Ethyl-1-hexanol	131.5 ± 24.21	129.0 ± 24.51	95.27 ± 4.130	-	-
70	46.98	1-Octanol	-	-	80.54 ± 12.42	-	-
*Acids*							
83	53.51	3-Methylbutanoic acid	418.9 ± 48.21	437.0 ± 77.70	-	297.7 ± 46.61	95.85 ± 16.71
94	59.90	(Pyrrol-3-yl) acetic acid	-	182.2 ± 15.59	93.53 ± 9.126	276.5 ± 45.85	115.9 ± 7.289
95	60.13	3-Methyl-2-butenoic acid	59.71 ± 5.449	105.8 ± 9.025	-	132.0 ± 11.38	73.68 ± 11.98
*Others*							
7	13.49	2,2,4,6,6-Pentamethylheptane	15.3 ± 1.552	-	-	-	-
9	15.30	Decane	31.96 ± 3.145	16.74 ± 1.903	8.31 ± 0.4633	-	-
16	21.14	2,3,6-Trimethyl-1,5-heptadiene	-	28.64 ± 3.653	21.54 ± 2.496	-	19.32 ± 2.044
20	23.52	*p*-Xylene	20.14 ± 2.173	-	-	-	-
26	26.14	Dodecane	53.13 ± 4.700	127.3 ± 14.52	64.57 ± 6.056	62.26 ± 12.11	-
27	26.33	Trimethyloxazole	25.72 ± 4.273	39.29 ± 2.386	43.27 ± 7.174	-	47.78 ± 5.174
37	30.61	Styrene	-	53.67 ± 0.409	33.55 ± 3.740	31.48 ± 1.251	32.17 ± 3.835
40	31.71	2,7-Dimethyloxepine	-	58.62 ± 7.032	31.20 ± 1.046	-	-
41	32.34	3-Methylanisole	-	252.9 ± 20.84	133.1 ± 3.533	111.7 ± 16.03	81.05 ± 5.853
79	51.04	1-Acetyl-2-methylcyclopentene	108.3 ± 18.89	327.0 ± 48.98	164.3 ± 21.58	265.4 ± 28.01	136.5 ± 12.05
91	57.86	Cyclopentene	45.19 ± 4.663	-	-	-	-

RT: retention time; -: Not detected.

**Table 2:** Potential properties of some important volatile organic metabolites (VOMs) identified in spent coffee grounds SCGs analyzed in this study.

VOMs	Antidiabetic	Anti-inflammatory	Antimicrobial	Antioxidant	Antiproliferative	Antitumor	Cytotoxic	Flavors	References
Furfural		x	x	x					[25,27,28,29,30,31,32,33,34,35]
5-Methylfurfural			x			x		x	
2-Furanmethanol		x	x	x					
2-Ethyl-6-methyl-pyrazine								x	
3-Ethyl-2,5-dimethylpyrazine								x	
Pyrrole-2-carboxaldehyde			x						
Hexanal			x						
β-Myrcene			x	x			x		
Limonene	x	x	x	x	x	x	x		
Carvacrol		x	x	x	x	x	x		
Menthene		x	x	x					
Dimethyl disulfide				x					
Guaiacol			x	x					

## Data Availability

The data presented in this study are available on request from the corresponding author.

## Supplementary Materials

The following supporting information can be downloaded at: https://www.mdpi.com/article/10.3390/foods11121731/s1, Figure S1: Chromatograms obtained through HS-SPME/GC-MS for spent coffee grounds from different geographical origin (attribution of the peak number is shown in Table 1).
