# Supplementary material for: Valorization of Spent Coffee Grounds as a Natural Source of Bioactive Compounds for Several Industrial Applications—A Volatilomic Approach
Source: Foods. 2022 Jun 13;11(12):1731. doi: 10.3390/foods11121731 (PMC9222233; doi:10.3390/foods11121731)
Supplement: Supplementary file 1 [file foods-11-01731-s001.zip › foods-1753087-supplementary.pdf]

Article

Valorization of spent coffee grounds as a natural source of bioactive compounds for several industrial applications. A volatilomic approach

Carolina Andrade [1], Rosa Perestrelo [1,*] and José S. Câmara [1,2,]

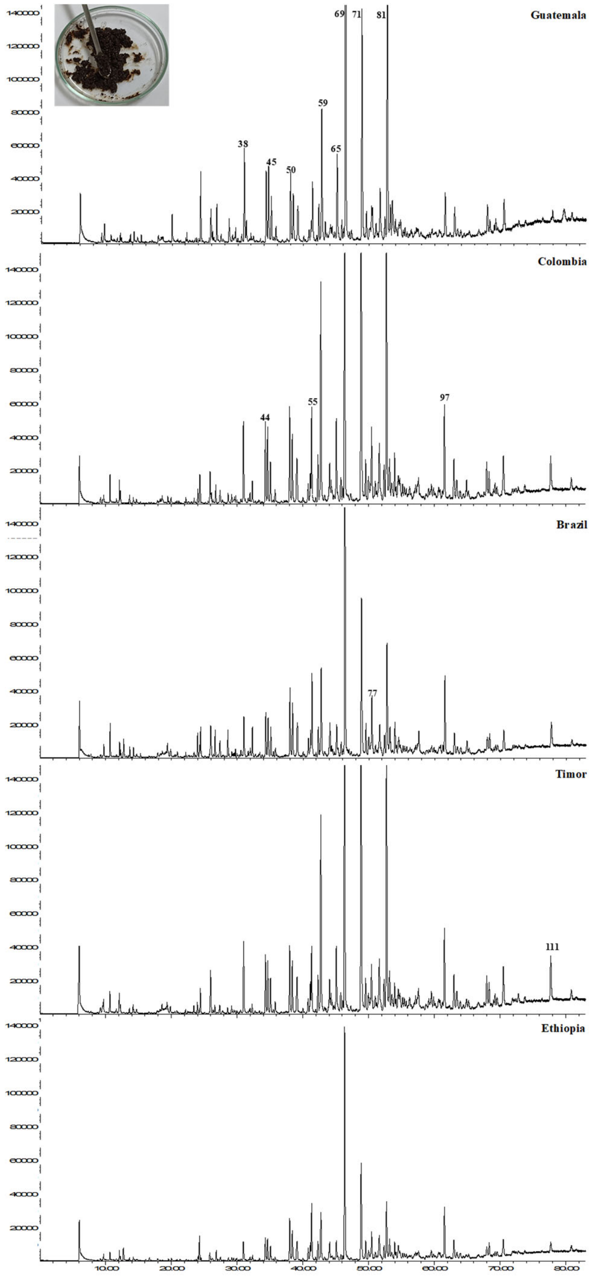

Figure S1. Chromatograms obtained through HS-SPME/GC-MS for spent coffee grounds from different geographical origin (attribution of the peak number is shown in Table 1).
